# Impact of adaptive intensity-modulated radiotherapy on the neutrophil-to-lymphocyte ratio in patients with nasopharyngeal carcinoma

**DOI:** 10.1186/s13014-019-1350-9

**Published:** 2019-08-22

**Authors:** Ning Han, Xintong Lyu, Guang Li, Qiao Qiao

**Affiliations:** Department of Radiation Oncology, the First Hospital of China Medical University, Shenyang, 110001 Liaoning China

**Keywords:** Adaptive radiotherapy, Nutritional status, Haematological parameters, Side effects

## Abstract

**Purpose:**

Nutritional status and haematological parameters are related to the prognosis of patients treated with radiotherapy, but the correlation between adaptive radiotherapy (ART) and haematological indicators has never been reported. This study explores the influence of ART on the change in haematological indicators and provides a theoretical basis for the use of ART in patients with nasopharyngeal carcinoma (NPC).

**Patients and methods:**

We retrospectively analysed 122 patients with NPC from January 2014 to December 2015. Patients in two treatment groups were matched using the propensity score matching method at a ratio of 1:1. The data were analysed with the Kaplan–Meier method, log-rank tests, regression analyses and paired t tests.

**Results:**

Significant differences were detected for changes in the neutrophil-to-lymphocyte ratio (ΔNLR), circulating lymphocyte count (ΔCLC), circulating platelet count (ΔCPC), and circulating neutrophil granulocyte count (ΔCNC) during radiotherapy (*P* = 0.002, *P* < 0.001, and *P* = 0.036, respectively) between the ART and non-ART groups. Differences in acute radiation injury to the parotid glands (PGs) (*P* < 0.001), skin (P < 0.001), and oral structures (P < 0.001), Δweight (kg) (*P* = 0.025), and Δweight (%) (*P* = 0.030) were also significant between the two groups. According to univariate and multivariate analyses, ART (R = 0.531, *P* = 0.004), skin-related side effects (R = 0.328, *P* = 0.020), and clinical stage (R = -0.689, *P* < 0.001) are influencing factors for the ΔNLR in patients. ART is also the influencing factor for the ΔCLC (R = 2.108, *P* < 0.001) and the only factor affecting the ΔCPC (R = 0.121, *P* = 0.035). Based on subgroup analyses, for stage T1–2N0–3 disease, ΔCLC was higher in patients in the ART group than in patients in the non-ART group (*P* < 0.001, *P* = 0.003, and P = 0.003).

**Conclusion:**

ART ameliorates changes in haematological indexes (ΔNLR, ΔCLC, and ΔCPC) and reduces side effects to the skin and PGs and weight loss during radiotherapy in patients with NPC, and patients with stage T1–2 disease experience a greater benefit.

**Electronic supplementary material:**

The online version of this article (10.1186/s13014-019-1350-9) contains supplementary material, which is available to authorized users.

## Introduction

Intensity-modulated radiotherapy (IMRT) is the main treatment that facilitates the delivery of high radiation doses to the target and reduces the delivered dose to organs. IMRT shows excellent local control with few toxicities [1]. However, during IMRT, a significant shrinkage of the tumours and weight loss may occur in patients with nasopharyngeal carcinoma (NPC), and these changes can result in the delivery of decreased radiation doses to the tumour and increased doses to normal tissues [2]. Adaptive radiotherapy (ART) instantly corrects the target and dose based on repeat computed tomography (CT) imaging from each patient and re-planning during the course of IMRT to identify dosimetric changes and ensure the delivery of adequate doses to target volumes and safe doses to normal tissues; thus, ART can significantly alleviate late effects (injury to the mucosa and xerostomia) in patients [3, 4].

Studies show that 30–60% of patients with head and neck cancer (HNC) suffer from malnutrition caused by complex factors, including swallowing pain, anorexia and radiotherapy-induced symptoms, all of which impair the patient’s ability to eat, and many patients lose additional weight during and after treatment [5, 6]. These factors greatly aggravate malnutrition of patients during radiotherapy; additionally, poor nutritional status is significantly associated with poor prognosis in patients with head and neck squamous cell carcinoma [7–9]. ART can limit oral side effects and xerostomia resulting from radiation-induced damage mainly to the parotid glands (PGs) [4, 10], thereby enhancing the nutritional intake of patients and improving nutrition during and after radiotherapy.

With the rising incidence of HNC, the TNM staging system remains inadequate, and it is becoming increasingly important to find reliable prognostic parameters [11, 12]. Many studies have suggested that haematological parameters such as platelet counts and the neutrophil-to-lymphocyte ratio (NLR) can be used as indicators to predict the prognosis of cancer patients [8, 13–19]. Several pretherapeutic laboratory values, such as red cell count, have prognostic relevance for overall survival (OS) in patients with HNC [20].

Lou Y et al. found that compared with IMRT alone, IMRT re-planning facilitates improved local–regional recurrence-free survival (LRFS) in patients with stage T3/T4 NPC [21]. Several studies had previously proposed that nutritional status and haematological parameters during radiotherapy are associated with prognosis in patients with head and neck squamous cell carcinoma [7, 8, 13, 14]. In recent years, studies have suggested that nutritional status and haematological parameters are related to prognosis, but the correlation between ART and haematological indicators, such as the NLR, has never been reported. This study intends to explore the influence of ART on the change in haematological indicators and provide a theoretical basis for the use of ART in patients with NPC.

## Material and methods

### Patients

**Fig. 1:**
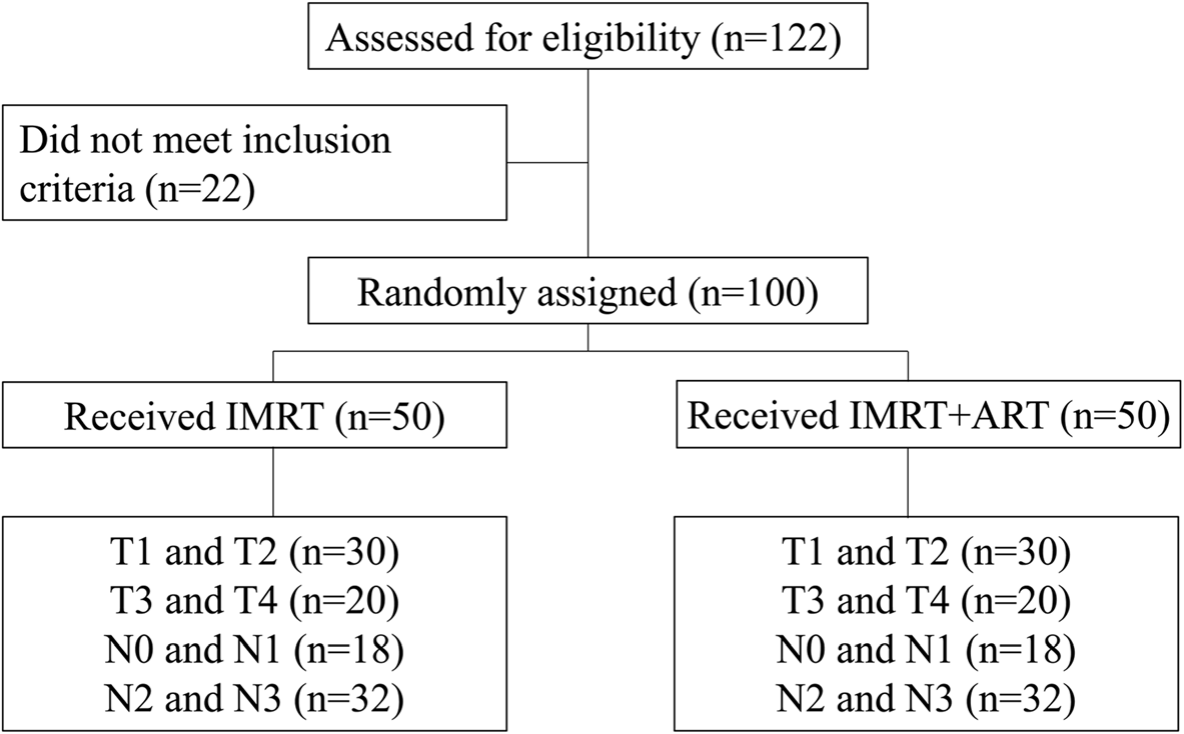
Overall study flow chart. Abbreviations: IMRT,Intensity-modulated radiotherapy; ART, adaptive radiotherapy

We included 122 newly diagnosed patients with histologically confirmed nonmetastatic NPC, who received radical radiotherapy with or without concurrent chemotherapy (CCT) in our hospital between January 2014 and December 2015 (Fig. 1). All patients were staged according to the 8th edition of the American Joint Committee on Cancer (AJCC) staging system [22]. This study was approved by the ethics committee of our hospital. Informed consent was obtained.

### IMRT

All patients received IMRT with 6-megavoltage (MV) photons. The gross tumour target of the nasopharynx (GTVnx) and involved lymph nodes (GTVln) were outlined based on CT and magnetic resonance imaging (MRI) scans. The clinical target volume 1 (CTV1) included the GTVnx with a 5–10 mm margin and high risk structures. The clinical target volume 2 (CTV2) included regions of the nasopharyngeal cavity, maxillary sinus, pterygopalatine fossa, posterior ethmoid sinus, parapharyngeal space, skull base, anterior third of clivus, inferior sphenoid sinus, and cavernous sinus. The clinical lymph node volume (CTVln) included the upper neck lymphatic drainage regions. Organs at risk (OAR) were also outlined. The contoured critical structures included the brain stem, chiasm, optic nerves, spinal cord, eyes, lens, PGs, oral cavity, larynx, mandible, and temporomandibular joints. The prescribed doses were defined as follows: 66–70 Gy for GTVnx and GTVln; 60 Gy for CTV1; and 50 Gy for CTV2. Each dose was divided into 28–33 fractions. The dose limits for normal organs were set according to the Radiation Therapy Oncology Group (RTOG) protocol 0225 [23].

### Art

**Fig. 2:**
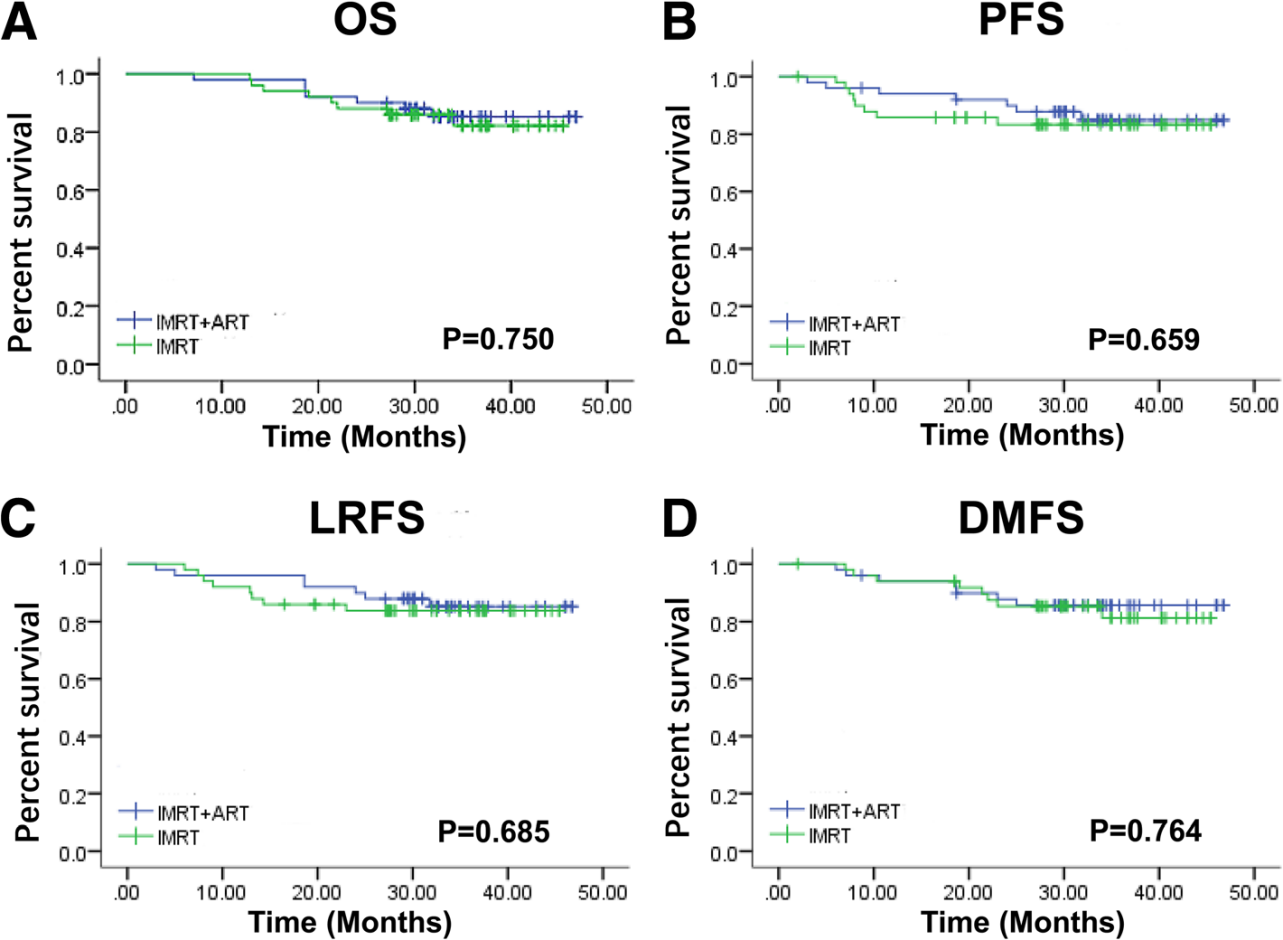
Survival analysis of the IMRT group and the ART+IMRT group. Abbreviations: OS, overall survival; LRFS, local–regional recurrence-free survival; DMFS, distant metastasis-free survival;PFS, progression-free survival

All patients underwent weekly CT scanning. During each repeat CT scan, the patient maintained the same position, and the new CT scan was used to generate a new IMRT plan for the corresponding fractions of treatment. During the treatment, if re-planning was necessary, the target and OAR were re-contoured as required on the repeat CT scan and a new plan generated (Fig. 2). The aim of the new plan was to achieve comparable target volume coverage and OAR doses to the original plan.

### CCT

CCT, which included cisplatin (75 mg/m2, days 1–3), was given to all patients.

### Haematological parameters

Five parameters, namely, changes during radiotherapy in the NLR (ΔNLR), the circulating lymphocyte count (ΔCLC), the circulating platelet count (ΔCPC), the circulating neutrophil granulocyte count (ΔCNC), and the haemoglobin count (ΔHB), were analysed. The ΔNLR during radiotherapy is the result of subtraction of the count before radiotherapy (NLR1) from the count after radiotherapy (NLR2) divided by the count before radiotherapy (NLR1): ΔNLR = NLR2-NLR1/NLR1. The ΔCLC during radiotherapy is the result of subtraction of the CLC before radiotherapy (CLC1) from the CLC after radiotherapy (CLC2) divided by the CLC before radiotherapy (CLC1): ΔCLC = (CLC2-CLC1)/CLC1. The ΔCNC during radiotherapy is the result of subtraction of the CNC before radiotherapy (CNC1) from the CNC after radiotherapy (CNC2) divided by the CNC before radiotherapy (CNC1): ΔCNC = (CNC2-CNC1)/CNC1. The ΔCPC during radiotherapy is the result of subtraction of the CPC before radiotherapy (CPC1) from the CPC after radiotherapy (CPC2) divided by the CPC before radiotherapy (CPC1): ΔCPC = (CPC2-CPC1)/CPC1. The ΔHB during radiotherapy is the result of the subtraction of HB before radiotherapy (HB1) from HB after radiotherapy (HB2) divided by HB before radiotherapy (HB1): ΔHB = (HB2-HB1)/HB1.

### Follow-up

All patients were evaluated weekly during radiotherapy and examined in follow-up appointments that were scheduled up to 1 month after the completion of radiotherapy and then every 3 months in years 1–2, every 6 months in years 3–5, and annually thereafter. Each follow-up included a flexible fibreoptic endoscopy, abdominal ultrasound, chest X-ray and basic serum chemistry. Either CT or MRI of the head and neck was also performed after the completion of IMRT and every 6 months thereafter.

### Statistics

Propensity score matching was used to divide the patients into two groups (IMRT and ART+IMRT). A one-to-one matching without replacement was performed using a 0.5 caliper width. The χ2 test and paired t test were used to test the baseline balance between the two groups. The relationship between the change in haematological parameters and treatment-related factors was analysed by Pearson’s correlation. Variables with *P* < 0.05 were included in a multivariate analysis, performed by regression analysis. Subgroup analyses were performed using paired t tests. The rates of LRFS, distant metastasis-free survival (DMFS), progression-free survival (PFS) and OS were estimated with the Kaplan–Meier method and compared with the log-rank test. All data were analysed using SPSS 22.0 software package (IBM Corporation, Armonk, NY, USA).

## Results

### Patients and characteristics

After matching, 55 and 67 patients were treated with ART+IMRT and IMRT, respectively. Among them, 50 patients treated with ART+IMRT and 50 patients with IMRT were included in the analysis. All subsequent analyses were based on the propensity-matched cohort. The characteristics of patients after propensity score matching are shown in Table 1.

**Table 1:** Patient characteristics and treatment details after propensity score matching

Characteristics	ART+IMRT (*n* = 50)	IMRT (n = 50)	P
Age (years)	54 (16~72)	55 (22~73)	0.865
Gender			0.500
Male	33 (66%)	34 (68%)	
Female	17 (34%)	16 (32%)	
History of smoking			0.500
Yes	22 (44%)	23 (46%)	
No	28 (56%)	27 (54%)	
History of drinking			0.795
Yes	8 (16%)	10 (20%)	
No	42 (84%)	40 (80%)	
Family history			0.795
Yes	10 (20%)	8 (16%)	
No	40 (80%)	42 (84%)	
T stage			1
T1	1 (2%)	1 (2%)	
T2	29 (58%)	29 (58%)	
T3	14 (28%)	14 (28%)	
T4	6 (12%)	6 (12%)	
N stage			1
N0	7 (14%)	7 (14%)	
N1	11 (22%)	11 (22%)	
N2	27 (54%)	27 (54%)	
N3	5 (10%)	5 (10%)	
Clinical stage			1
II	3 (6%)	3 (6%)	
III	40 (80%)	40 (80%)	
IV	7 (14%)	7 (14%)	
CCT	50 (100%)	50 (100%)	1

*Abbreviations*: *IMRT* Intensity-modulated radiotherapy, *ART* adaptive radiotherapy, *CCT* concurrent chemotherapy

### Changes in haematological parameters in different radiotherapy modes

There were no significant differences in the pre-treatment NLR, CLC, CPC, or CNC between the two groups, while the differences in the ΔNLR (*P* = 0.002), ΔCLC (*P* < 0.001), and ΔCPC (*P* = 0.036) were statistically significant. Differences in acute radiation injury classification of the PGs (P < 0.001), skin (P < 0.001) and oral structures (P < 0.001) were also significant in both groups and are shown in Table 2. We also compared Δweight (kg) and Δweight (%) for the two groups during radiotherapy and observed a significant difference (*P* = 0.025 and *P* = 0.030, respectively).

**Table 2:** Comparison of radiotherapy-related variables between the two groups

Characteristics	ART+IMRT(n = 50)	IMRT(n = 50)	P
Pre-NLR	2.00 ± 1.78	2.81 ± 2.54	0.076
Pre-CLC(10^9^/L)	1.54 ± 0.60	1.66 ± 0.76	0.381
Pre-CNC(10^9^/L)	4.12 ± 1.65	4.16 ± 1.87	0.907
Pre-CPC(10^9^/L)	222.9 ± 63.02	239.8 ± 59.93	0.155
Pre-HB(g/L)	143.26 ± 15.46	138.92 ± 44.15	0.534
ΔNLR(%)	−1.80 ± 114.02	70.34 ± 49.40	< 0.001^*^
ΔCLC(%)	− 126.33 ± 119.97	−341.36 ± 320.99	< 0.001^*^
ΔCNC(%)	−14.43 ± 65.43	−30.57 ± 89.83	0.288
ΔCPC(%)	−4.52 ± 23.15	−16.62 ± 32.07	0.036^*^
ΔHCG(%)	45.68 ± 11.19	−45.7 ± 26.88	0.303
Δweight (Kg)	−3.76 ± 3.13	−5.04 ± 2.09	0.025^*^
ΔWeight(%)	−7.99 ± 3.60	−5.98 ± 4.87	0.030^*^
Acute radiation injury classification			
Parotid glands			< 0.001^*^
0	0	0	
1	13	0	
2	37	50	
3	0	0	
4	0	0	
Skin			< 0.001^*^
0	2	0	
1	38	18	
2	9	26	
3	1	6	
4	0	0	
Oral			< 0.001^*^
0	8	0	
1	0	14	
2	28	12	
3	14	24	
4	0	0	

*Abbreviations*: *NLR* neutrophil-to-lymphocyte ratio, *CLC* circulating lymphocyte count, *CPC* circulating platelet count, *CNC* circulating neutrophil granulocyte count, *HB* the haemoglobin count

^*^P ≤ 0.05

Side effects were significantly different between the two groups; however a previous study had concluded that the mean dose (Dmean) to the PGs is related to xerostomia during the course of IMRT and that ART can decrease the Dmean to the PGs [10]. Therefore, we compared Dmean and dose to 50% of the volume (D50) for the PGs and Dmean, D50, and the maximum dose for the skin. Additionally, we excluded 19 patients with missing weekly CT data in the IMRT group, and 19 matched patients in the ART+IMRT group were also excluded. Moreover, 1 patient without weekly CT data in the ART+IMRT group was excluded, and the matched patient in the IMRT group was also excluded. Hence, the new IMRT (IMRTnew) and the new ART+IMRT (ART+IMRTnew) groups were created, with 30 patients in each group. By comparison, differences in the Dmean, and D50 for the PGs and D50 for the skin were significant. For ipsilateral PGs, the difference in D50 and Dmean between the two groups was 1.28 Gy (*P* = 0.021) and 1.12 Gy (*P* = 0.038), respectively. For contralateral PGs, the difference in D50 and Dmean between the two groups was 1.28 Gy (P = 0.021) and 0.92 Gy (*P* = 0.034), respectively. For the skin, the difference in D50 between the two groups was 11.85 Gy (*P* < 0.001) (Table 3).

**Table 3:** Dosimetric changes in the OAR

OARs	ART+IMRT new No.Gy	IMRT new No.Gy	P
PG-ips			
D50	26.11 ± 6.12	27.39 ± 7.19	0.021^*^
Dmean	28.68 ± 5.99	29.80 ± 7.12	0.038^*^
PG-con			
D50	24.69 ± 3.92	25.95 ± 4.20	0.009^*^
Dmean	27.10 ± 4.01	28.02 ± 4.38	0.034^*^
Skin			
D50	3.03 ± 3.18	14.88 ± 6.65	< 0.001^*^
Dmean	21.22 ± 22.27	18.52 ± 4.21	0.491
Dmax	70.64 ± 5.52	71.29 ± 5.41	0.076

*Abbreviations*: *OAR* organs at risk, *PGs* parotid glands, *Dmean* mean dose, *D50* dose to 50% of the volume, *IMRT new* the new IMRT, *ART+IMRT* new,the new ART+IMRT

^*^P ≤ 0.05

### Changes in haematological parameters and related factors

Relevant variables were included in the correlation analysis and regression analysis. The ΔNLR, ΔCLC, and ΔCPC were normally distributed. In univariate analyses, clinical stage (R = -0.719, *P* = 0.001), ART (R = -0.721, *P* < 0.001) and acute radiation injury grade of the skin (R = 0.536, P = 0.001) were significantly associated with the ΔNLR. Additionally, ART was significantly associated with the ΔCLC (R = 2.150, P < 0.001), and ART (R = 0.121, *P* = 0.035) was the only significantly correlated factor with the ΔCPC (Table 4).

**Table 4:** Univariate analysis of the ΔNLR, ΔCLC, and ΔCPC

Characteristics	ΔNLR				ΔCLC				ΔCPC			
	Regression coefficient	SD	P	Pearson coefficient	Regression coefficient	SD	P	Pearson coefficient	Regression coefficient	SD	P	Pearson coefficient
Age (years)	0.003	0.009	0.757	0.310	0.002	0.025	0.942	0.007	−0.003	0.003	0.333	−0.098
Gender	−0.225	0.203	0.270	−0.111	−0.782	0.564	0.169	−0.139	−0.024	0.061	0.702	− 0.039
History of smoking	0.202	0.193	0.297	0.105	−0.067	0.543	0.902	−0.013	− 0.067	0.058	0.253	−0.115
History of drinking	0.221	0.248	0.375	0.090	0.375	0.696	0.591	0.054	0.050	0.075	0.503	0.068
Family history	−0.354	0.247	0.155	−0.143	0.189	0.697	0.787	0.027	−0.031	0.075	0.679	−0.042
T stage	−0.243	0.129	0.064	−0.186	0.593	0.363	0.105	0.163	0.017	0.040	0.662	0.044
N stage	−0.015	0.119	0.902	−0.013	−0.208	0.333	0.535	−0.063	0.028	0.036	0.435	0.079
Clinical stage	−0.719	0.205	0.001^*^	−0.334	1.023	0.600	0.091	0.170	−0.015	0.066	0.816	−0.024
ART	−0.721	0.177	< 0.001^*^	−0.380	2.150	0.490	< 0.001^*^	0.406	0.121	0.057	0.035^*^	0.211
Acute radiation injury classification												
Parotid glands	0.426	0.282	0.133	0.151	−0.594	0.791	0.456	−0.075	− 0.077	0.086	0.370	−0.091
Skin	0.536	0.136	< 0.001^*^	0.370	−0.785	0.401	0.053	−0.194	−0.046	0.044	0.293	−0.106
Oral	0.110	0.104	0.296	0.106	0.331	0.291	0.258	0.114	−0.001	0.032	0.978	−0.003

*Abbreviations*: *ART* adaptive radiotherapy, *NLR* neutrophil-to-lymphocyte ratio, *CLC* circulating lymphocyte count, *CPC* circulating platelet count

^*^P ≤ 0.05

According to the results of univariable analyses, ART, acute radiation injury grade of the skin, and clinical stage were included in a multivariate analysis. Based on the results of the multivariate analysis, ART, acute radiation injury grade of the skin, and clinical stage were the influencing factors of the ΔNLR in patients (R = 0.531, *P* = 0.004; R = 0.328, *P* = 0.020; and R = -0.689, *P* < 0.001, respectively). ART was further included in a multivariate regression analysis of ΔCLC. Because acute radiation injury grade of the skin had a *P* value of 0.053, close to 0.05, this factor was also included in the multivariate analysis. After calculation, the side effect to the skin was excluded, and ART was identified as the influencing factor of the ΔCLC (R = 2.108, *P* < 0.001). ART was also the only factor that affected the ΔCPC (R = 0.121, *P* = 0.035) (Table 5).

**Table 5:** Multivariate analysis of the ΔNLR, ΔCLC, and ΔCPC

Characteristics	ΔNLR					ΔCLC					ΔCPC				
	Regression coefficient	SD	Standard regression coefficient	T	P	Regression coefficient	SD	Standard regression coefficient	T	P	Regression coefficient	SD	Standard regression coefficient	T	P
ART	− 0.531	0.181	− 0.280	−2.933	0.004^*^	2.108	0.549	0.398	3.842	< 0.001^*^	0.121	0.057	0.211	2.141	0.035^*^
Acute injury of skin	0.328	0.139	0.226	2.366	0.020^*^	−0.073	0.419	−0.018	−0.174	0.862	–	–	–	–	–
Clinical stage	−0.689	0.185	−0.320	−3.727	< 0.001^*^	–	–	–	–	–	–	–	–	–	–

*Abbreviations*: *ART* adaptive radiotherapy, *NLR* neutrophil-to-lymphocyte ratio, *CLC* circulating lymphocyte count, *CPC* circulating platelet count

^*^P ≤ 0.05

### Subgroup analysis

To identify patients who benefited the most from ART, we performed subgroup analyses according to the T stage (T1–2 and T3–4) and N stage (N0–2 and N3). The clinical stage was related to the ΔNLR during radiotherapy and was identified as an independent prognostic factor of the ΔNLR. However, there was not a wide distribution among clinical stages, and TNM staging is closely related to the clinical stage. Therefore, we performed subgroup analysis according to the TNM classification.

For patients with stage T1-4 N0–3 disease, the ΔNLR was higher in patients treated with IMRT than in patients treated with ART+IMRT (*P* = 0.018, *P* = 0.032, *P* = 0.029, and *P* = 0.004, respectively). For patients with T1–2N0–3 disease, the ΔCLC was higher in patients treated with ART+IMRT than in patients treated with IMRT (*P* < 0.001, *P* = 0.003, and P = 0.003, respectively). These differences were significant (Table 6).

**Table 6:** Comparison of the ΔNLR, ΔCLC, and ΔCPC in different subgroups according to TNM stages

Characteristics	ΔNLR(%)			ΔCLC(%)			ΔCPC(%)		
	ART+IMRT	IMRT	P	ART+IMRT	IMRT	P	ART+IMRT	IMRT	P
T stage									
1–2(*n* = 30)	15.61 ± 65.69	59.03 ± 58.52	0.018^*^	−117.93 ± 88.14	−406.47 ± 350.15	< 0.001^*^	−6.80 ± 20.87	−19.07 ± 34.61	0.107
3–4(*n* = 20)	−27.92 ± 157.78	57.65 ± 34.57	0.032^*^	−138.93 ± 155.13	−243.70 ± 240.36	0.095	−1.1 ± 26.93	−12.96 ± 29.24	0.199
N stage									
0–1(*n* = 18)	2.17 ± 130.78	76.54 ± 12.69	0.029^*^	−109.18 ± 107.03	− 312.87 ± 219.52	0.003^*^	−6.21 ± 24.70	−18.57 ± 25.47	0.171
2–3(*n* = 32)	−4.03 ± 107.59	66.85 ± 61.10	0.004^*^	− 135.98 ± 129.10	− 357.39 ± 372.84	0.003^*^	−3.58 ± 22.97	−15.53 ± 36.05	0.117

*Abbreviations*: *ART* adaptive radiotherapy, *IMRT* Intensity-modulated radiotherapy, *NLR* neutrophil-to-lymphocyte ratio, *CLC* circulating lymphocyte count; CPC, circulating platelet count

^*^P ≤ 0.05

### Survival

**Fig. 3:**
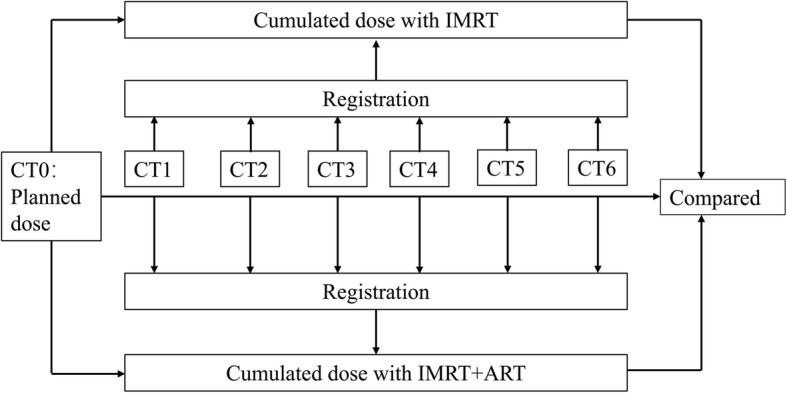
ART flow chart. Weekly CT scans were performed during the treatment. Doses were calculated for each weekly fraction. Abbreviations: IMRT, Intensity-modulated radiotherapy; ART, adaptive radiotherapy; NPC, nasopharyngeal carcinoma; CT, computed tomography

The median follow-up time in the IMRT group was 33 months (12.9–45.4 months), and that in the ART+IMRT group was 33.1 months (7.1–46.8 months). The differences in OS(Fig. 3a), PFS(Fig. 3b), LRFS(Fig. 3c), and DMFS(Fig. 3d) between these two groups were not statistically significant (*P* = 0.750, *P* = 0.659, *P* = 0.685 and *P* = 0.764, respectively) (Fig. 3). Local recurrence was found in 3 (6%) patients in the ART+IMRT group and in 9 (18%) patients in the IMRT group, with fewer local recurrences in the ART+IMRT group than in the IMRT group.

## Discussion

In this study, we found that ART reduces side effects during radiotherapy in patients with NPC. We compared the acute radiation injury responses of the PGs, skin and oral structures in the last week of radiotherapy, and the differences were obvious. The differences in the ART+IMRT group was significantly less pronounced than that in the IMRT group. The acute radiation response grade for the PGs was ≥2 after the treatment in 50 patients receiving IMRT, while this was true for 37 patients in the ART+IMRT group; likewise, the acute radiation response grade for the skin was ≥2 for 32 patients in the IMRT group and 10 patients in the ART+IMRT group. The reason was that ART decreased the radiation dose to normal tissues [3, 4]. The PGs are the most vulnerable organ during radiotherapy, regardless of volume or displacement, and the dose to the PGs results in the risk of xerostomia. Excess radiation dose to the PGs increases the risk of xerostomia, leading to a deterioration in the quality of life (QOL) [10]. Severe side effects, such as xerostomia and difficulty in swallowing can lead to malnutrition, which is associated with poor prognosis [10, 24]. Joel Castelli et al. noted that anatomical changes during IMRT were the main cause of overdose to the PGs [10]. The average volume of the PGs decreased by 28% during radiotherapy, and the weekly re-planning could account for the changes in the volume of the PGs in real time. The Dmean for the PGs decreased by an average of 5 Gy, and the risk of xerostomia decreased by 11% [10]. The Dmean to the PGs is associated with the volume of PGs during radiotherapy [25–30], and there was a difference between the delivered dose and the planned dose to the PGs during radiotherapy. Brouwer also demonstrated that the Dmean to the PGs in patients with HNC significantly increased during radiotherapy, and ART reduced the Dmean to the PGs, thus alleviating the symptoms of xerostomia [4]. Deng et al. noted that patients with NPC who received IMRT experienced significant anatomical changes during the course of treatment, and ART was necessary to maintain optimal doses to targets and OAR. Patients with NPC who were subjected to re-planning at cycles 5 and 15 were compared with patients who only received IMRT. The planning target volume in the ART group were significantly improved. Compared with those in the IMRT group, the Dmean to the PGs in the ART+IMRT group decreased by 1.27 ± 1.05 Gy, the V50 to the PGs decreased by 4.12 ± 3.58%, and the V55 for the skin decreased by 0.91 ± 1.83% [31].

Myelosuppression is a common side effect of radiotherapy. When myelosuppression occurs, haematopoietic stem cells cannot produce enough normal blood cells, which leads to complications such as anaemia, infection and bleeding, and these complications seriously affect the survival of patients. Moreover, as inflammation plays an important role in tumour development [32], many studies have suggested that inflammation-related factors (such as the NLR, lymphocyte count, and neutrophil count) in the blood can predict the prognosis of patients, [32–39] and these parameters can be evaluated by conventional examinations. Several studies have shown that a decrease in lymphocyte count in the peripheral blood of patients with NPC is associated with poor OS and PFS [33, 34, 36, 37], and that the neutrophil count is related to OS, DSS, and DMFS [33, 38]. The NLR was also thought to be associated with prognosis in many types of cancers [39–41], and a higher NLR is related to poor prognosis [42–47]. A meta-analysis by Yukinori Takenaka demonstrated that elevated NLR was associated with poor OS, DSS, PFS, and DMFS [48]. In our study, 12 (24%) patients treated with ART+IMRT had a lower NLR after treatment than that before; additionally, 41 (81%) patients experienced a decrease in the CLC, 36 (72%) patients experienced a decrease in the CNC, and 31 (62%) patients experienced a decrease in the CPC after treatment. For the IMRT group, these number were 3 (6%), 48 (96%), 24 (48%), and 38 (76%), respectively. Patients in the ART and IMRT groups demonstrated significant differences in the ΔNLR (*P* < 0.001), ΔCLC (P < 0.001), ΔCNC (*P* = 0.045), and ΔCPC (*P* = 0.03). This finding supports the changes in haematological parameters during radiotherapy in patients. ART can ameliorate the decrease in haematological parameters during radiotherapy and mitigate myelosuppression after radiotherapy.

Furthermore, correlation analysis and regression analysis showed that ART was an independent factor influencing haematological parameters (ΔNLR, ΔCLC, and ΔCPC). A study of cervical cancer patients by Emily et al. suggested that pre-treatment total lymphocyte count (TLC) ≥1000 cells/mm3 and post-treatment TLC > 500 cells/mm3 indicated a 77% (hazard ratio (HR): 0.23; 95% confidence interval (CI): 0.05–1.03; *P* = 0.053) and 58% decrease in the risk of death (HR: 0.42; 95% CI: 0.12–1.46; *P* = 0.17), respectively [8]. Unfortunately, no such studies have recently been conducted for HNC.

A retrospective study in 2016 suggested that ART can improve the prognosis of patients with NPC. The study followed 132 NPC patients (66 receiving ART and 66 receiving IMRT), and the 5-year LRFS rate was higher in the IMRT re-planning group than in the IMRT only group (96.7 vs. 88.1%, *P* = 0.022). Distant metastasis remains the main pattern of treatment failure. A total of 21.2% patients in the IMRT re-planning group and 28.8% patients in the IMRT only group had distant metastasis [21]. Two previous studies have also suggested that ART improves clinical outcomes in patients with HNC, including improvements in local control and reductions in late side effects [3, 21]. However, based on our follow-up data, the differences in OS, PFS, DMFS, and LRFS were not statistically significant, although 3 (6%) patients in the ART+IMRT group 9 (18%) patients in the IMRT group experienced local recurrence. During the course of radiotherapy, most patients experienced anatomical changes, such as tumour shrinkage and weight loss, resulting in an insufficient dose to the target area [2], which greatly improved the LRFS rate of patients. ART can alleviate these anatomical changes to maintain a satisfactory dose to the target volumes [21].

Although studies have shown that patients with the same TNM stage may have different clinical outcomes [49–51], TNM stages remain the standard for predicting prognosis and stratification of patients in studies. In our study, patients were separated according to their TNM stage. The results showed that the change in haematological parameters of patients with stage T1–2 disease was significantly better than that of patients with stage T3–4 disease. Notably, the number of patients in this study was limited.

## Conclusion

Our study compared changes in side effects, haematologic parameters and weight during radiotherapy between patients receiving ART+IMRT and IMRT alone. We found that ART had an effect on the side effects and the change in haematologic parameters during radiotherapy, and patients with T1–2 disease experienced a greater benefit. According to the follow-up, the differences in OS, PFS, LRFS and DMFS were not statistically significant, but the number of local recurrences in the IMRT group was higher than that in the ART+IMRT group. Nevertheless, these results are preliminary and need to be validated.

## Additional file

Additional file 1: Patient characteristics and radiotherapy-related variables between the two groups. (XLSX 23 kb)

## Data Availability

Data used in this study can be found in the Additional file 1: Table S1.

## References

[1] Xiao W, Huang S, Han F (2011). Local control, survival, and late toxicities of locally advanced nasopharyngeal carcinoma treated by simultaneous modulated accelerated radiotherapy combined with cisplatin concurrent chemotherapy: long-term results of a phase 2 study. Cancer..

[2] Cheng H, Wu V, Ngan R (2012). A prospective study on volumetric and dosimetric changes during intensity-modulated radiotherapy for nasopharyngeal carcinoma patients. Radiother Oncol.

[3] Zhao L, Wan Q, Zhou Y, Deng X, Xie C, Wu S (2011). The role of replanning in fractionated intensity modulated radiotherapy for nasopharyngeal carcinoma. Radiother Oncol.

[4] Brouwer C, Steenbakkers R, van der Schaaf A (2016). Selection of head and neck cancer patients for adaptive radiotherapy to decrease xerostomia. Radiother Oncol.

[5] Ravasco P, Monteiro-Grillo I, Vidal P, Camilo M (2003). Nutritional deterioration in cancer: the role of disease and diet. Clin Oncol (R Coll Radiol).

[6] Cho Y, Roh J, Jung J (2013). Prediction of posttreament significant body weight loss and its correlation with disease-free survival in patients with oral squamous cell carcinomas. Nutr Cancer.

[7] Moon H, Roh J, Lee S (2016). Prognostic value of nutritional and hematologic markers in head and neck squamous cell carcinoma treated by chemoradiotherapy. Radiother Oncol.

[8] Wu E, Oduyebo T, Cobb L (2016). Lymphopenia and its association with survival in patients with locally advanced cervical cancer. Gynecol Oncol.

[9] Grossman S, Ellsworth S, Campian J (2015). Survival in patients with severe lymphopenia following treatment with radiation and chemotherapy for newly diagnosed solid tumors. J Natl Compr Cancer Netw.

[10] Castelli J, Simon A, Louvel G (2015). Impact of head and neck cancer adaptive radiotherapy to spare the parotid glands and decrease the risk of xerostomia. Radiat Oncol.

[11] Dahlstrom K, Calzada G, Hanby J (2013). An evolution in demographics, treatment, and outcomes of oropharyngeal cancer at a major cancer center: a staging system in need of repair. Cancer..

[12] Rios Velazquez E, Hoebers F, Aerts H (2014). Externally validated HPV-based prognostic nomogram for oropharyngeal carcinoma patients yields more accurate predictions than TNM staging. Radiother Oncol.

[13] Lin Y, Chang K, Lin Y, Chang T (2017). Pretreatment combination of platelet counts and neutrophil-lymphocyte ratio predicts survival of nasopharyngeal cancer patients receiving intensity-modulated radiotherapy. Onco Targets Ther.

[14] Sumner W, Stokes W, Oweida A (2017). Survival impact of pre-treatment neutrophils on oropharyngeal and laryngeal cancer patients undergoing definitive radiotherapy. J Transl Med.

[15] Zhu M, Feng M, He F, Han B (2018). Pretreatment neutrophil-lymphocyte and platelet-lymphocyte ratio predict clinical outcome and prognosis for cervical Cancer. Clin Chim Acta.

[16] Zhou YC, Chen LL, Xu HB, Sun Q, Zhang Q, Cai HF, Jiang H (2018). Aging-related prognosis analysis of definitive radiotherapy for very elderly esophageal cancer. Cancer Med.

[17] Wu CC, Li SH, Lu HI, Lo CM, Wang YM, Chou SY, Chen YH (2018). Inflammation-based prognostic scores predict the prognosis of locally advanced cervical esophageal squamous cell carcinoma patients receiving curative concurrent chemoradiotherapy: a propensity score-matched analysis. PeerJ.

[18] Pike Luke R.G., Bang Andrew, Mahal Brandon A., Taylor Allison, Krishnan Monica, Spektor Alexander, Cagney Daniel N., Aizer Ayal A., Alexander Brian M., Rahma Osama, Balboni Tracy, Ott Patrick A., Hodi F. Stephen, Schoenfeld Jonathan D. (2019). The Impact of Radiation Therapy on Lymphocyte Count and Survival in Metastatic Cancer Patients Receiving PD-1 Immune Checkpoint Inhibitors. International Journal of Radiation Oncology*Biology*Physics.

[19] Liu X, Liu Z, Lin E, Chen Y, Sun X, Zhou Z (2018). A cumulative score based on preoperative fibrinogen and the neutrophil-lymphocyte ratio to predict outcomes in resectable gastric cancer. Cancer Manag Res.

[20] Peter F, Wittekindt C, Finkensieper M, Kiehntopf M, Guntinas-Lichius O (2013). Prognostic impact of pretherapeutic laboratory values in head and neck cancer patients. J Cancer Res Clin Oncol.

[21] Luo Y, Qin Y, Lang J (2017). Effect of adaptive replanning in patients with locally advanced nasopharyngeal carcinoma treated by intensity-modulated radiotherapy: a propensity score matched analysis. Clin Transl Oncol.

[22] Edge S, Compton C (2010). The American joint committee on Cancer: the 7th edition of the AJCC cancer staging manual and the future of TNM. Ann Surg Oncol.

[23] Lee N, Harris J, Garden AS (2009). Intensity-modulated radiation therapy with or without chemotherapy for nasopharyngeal carcinoma: radiation therapy oncology group phase II trial 0225. J Clin Oncol.

[24] Chen W, Lai C, Lee T (2013). Scintigraphic assessment of salivary function after intensity-modulated radiotherapy for head and neck cancer: correlations with parotid dose and quality of life. Oral Oncol.

[25] Broggi S, Fiorino C, Dell'Oca I (2010). A two-variable linear model of parotid shrinkage during IMRT for head and neck cancer. Radiother Oncol.

[26] Reali A, Anglesio S, Mortellaro G (2014). Volumetric and positional changes of planning target volumes and organs at risk using computed tomography imaging during intensity-modulated radiation therapy for head-neck cancer: an "old" adaptive radiation therapy approach. Radiol Med.

[27] Sanguineti G, Ricchetti F, Thomas O, Wu B, McNutt T (2013). Pattern and predictors of volumetric change of parotid glands during intensity modulated radiotherapy. Br J Radiol.

[28] Sanguineti G, Ricchetti F, Wu B, McNutt T, Fiorino C (2015). Parotid gland shrinkage during IMRT predicts the time to xerostomia resolution. Radiat Oncol.

[29] Vásquez Osorio E, Hoogeman M, Al-Mamgani A, Teguh D, Levendag P, Heijmen B (2008). Local anatomic changes in parotid and submandibular glands during radiotherapy for oropharynx cancer and correlation with dose, studied in detail with nonrigid registration. Int J Radiat Oncol Biol Phys.

[30] Wang X, Lu J, Xiong X (2010). Anatomic and dosimetric changes during the treatment course of intensity-modulated radiotherapy for locally advanced nasopharyngeal carcinoma. Med Dosim.

[31] Deng S, Liu X, Lu H, et al. Three-phase adaptive radiation therapy for patients with nasopharyngeal carcinoma undergoing intensity-modulated radiation therapy: Dosimetric analysis. Technol Cancer Res Treat. 2017:1533034617709396. 10.1177/1533034617709396 PMC5762048 28511585

[32] He J, Shen G, Ren Z (2012). Pretreatment levels of peripheral neutrophils and lymphocytes as independent prognostic factors in patients with nasopharyngeal carcinoma. Head Neck..

[33] Jin Y, Ye X, He C, Zhang B, Zhang Y (2015). Pretreatment neutrophil-to-lymphocyte ratio as predictor of survival for patients with metastatic nasopharyngeal carcinoma. Head Neck..

[34] Su L, Zhang M, Zhang W, Cai C, Hong J (2017). Pretreatment hematologic markers as prognostic factors in patients with nasopharyngeal carcinoma: a systematic review and meta-analysis. Medicine (Baltimore).

[35] Jiang R, Cai X, Yang Z (2015). Elevated peripheral blood lymphocyte-to-monocyte ratio predicts a favorable prognosis in the patients with metastatic nasopharyngeal carcinoma. Chin J Cancer..

[36] Li J, Jiang R, Liu W (2013). A large cohort study reveals the association of elevated peripheral blood lymphocyte-to-monocyte ratio with favorable prognosis in nasopharyngeal carcinoma. PLoS One.

[37] Luo X, He W, Huang H (2015). Design of a prognostic score model for nasopharyngeal carcinoma. Head Neck.

[38] Ke L, Xiang Y, Xia W (2016). A prognostic model predicts the risk of distant metastasis and death for patients with nasopharyngeal carcinoma based on pre-treatment interleukin 6 and clinical stage. Clin Immunol.

[39] Templeton A, McNamara M, Šeruga B (2014). Prognostic role of neutrophil-to-lymphocyte ratio in solid tumors: a systematic review and meta-analysis. J Natl Cancer Inst.

[40] Sunakawa Yu, Yang Dongyun, Cao Shu, Zhang Wu, Moran Miriana, Astrow Stephanie H., Hsiang Jack, Stephens Craig, Tsuji Akihito, Takahashi Takehiro, Tanioka Hiroaki, Negoro Yuji, Takagane Akinori, Tani Satoshi, Yamaguchi Tatsuro, Eto Tetsuya, Fujii Masashi, Ichikawa Wataru, Lenz Heinz-Josef (2018). Immune-related Genes to Dominate Neutrophil-lymphocyte Ratio (NLR) Associated With Survival of Cetuximab Treatment in Metastatic Colorectal Cancer. Clinical Colorectal Cancer.

[41] Mirili Cem, Guney Isa Burak, Paydas Semra, Seydaoglu Gulsah, Kapukaya Tuba Korkmaz, Ogul Ali, Gokcay Serkan, Buyuksimsek Mahmut, Yetisir Abdullah Evren, Karaalioglu Bilgin, Tohumcuoglu Mert (2018). Prognostic significance of neutrophil/lymphocyte ratio (NLR) and correlation with PET–CT metabolic parameters in small cell lung cancer (SCLC). International Journal of Clinical Oncology.

[42] Ye S, Bai L (2018). Comparison and validation of the value of preoperative inflammation marker-based prognostic scores in resectable pancreatic ductal adenocarcinoma. Cancer management and research.

[43] García-Ortega D, Álvarez-Cano A, Sánchez-Llamas L (2018). Neutrophil/lymphocyte ratio is associated with survival in synovial sarcoma. Surg Oncol.

[44] De Felice F, Tombolini M, Abate G, et al. Prognostic significance of the neutrophil/lymphocyte ratio in patients with non-human papilloma virus-related oropharyngeal Cancer: a retrospective cohort study. Oncology. 2018:1–6. 10.1159/000492389 30212829

[45] He W, Hu W, Kong P (2018). Systemic neutrophil lymphocyte ratio and mismatch repair status in colorectal cancer patients: correlation and prognostic value. J Cancer.

[46] Nishijima Tomohiro F., Deal Allison M., Lund Jennifer L., Nyrop Kirsten A., Muss Hyman B., Sanoff Hanna K. (2019). Inflammatory markers and overall survival in older adults with cancer. Journal of Geriatric Oncology.

[47] Thio Quirina C. B. S., Goudriaan W. Alexander, Janssen Stein J., Paulino Pereira Nuno Rui, Sciubba Daniel M., Rosovksy Rachel P., Schwab Joseph H. (2018). Prognostic role of neutrophil-to-lymphocyte ratio and platelet-to-lymphocyte ratio in patients with bone metastases. British Journal of Cancer.

[48] Takenaka Y, Kitamura T, Oya R (2017). Prognostic role of neutrophil-lymphocyte ratio in nasopharyngeal carcinoma: a meta-analysis. PLoS One.

[49] Li G, Gao J, Tao Y (2012). Increased pretreatment levels of serum LDH and ALP as poor prognostic factors for nasopharyngeal carcinoma. Chin J Cancer.

[50] Lee A, Ng W, Chan L (2012). The strength/weakness of the AJCC/UICC staging system (7th edition) for nasopharyngeal cancer and suggestions for future improvement. Oral Oncol.

[51] Li X, Chang H, Xu B (2017). An inflammatory biomarker-based nomogram to predict prognosis of patients with nasopharyngeal carcinoma: an analysis of a prospective study. Cancer Med.

